# Analysis of ACE2 Gene-Encoded Proteins Across Mammalian Species

**DOI:** 10.3389/fvets.2020.00457

**Published:** 2020-07-03

**Authors:** Ying Cao, Yeping Sun, Xiaodong Tian, Zhihua Bai, Yue Gong, Jianxun Qi, Di Liu, Wenjun Liu, Jing Li

**Affiliations:** ^1^CAS Key Laboratory of Pathogenic Microbiology and Immunology, Institute of Microbiology, Chinese Academy of Sciences, Beijing, China; ^2^CAS Key Laboratory of Special Pathogens and Biosafety, Wuhan Institute of Virology, Chinese Academy of Sciences, Wuhan, China; ^3^College of Animal Sciences and Veterinary Medicine, Guangxi University, Nanning, China; ^4^Savaid Medical School, University of Chinese Academy of Sciences, Beijing, China; ^5^National Science Library, Chinese Academy of Sciences, Beijing, China; ^6^Center for Biosafety Mega-Science, Institute of Microbiology, Chinese Academy of Sciences, Beijing, China

**Keywords:** COVID-19, SARS-CoV-2, ACE2 gene, protein, mammals

## Abstract

Human beings are currently experiencing a serious public health event. Novel coronavirus disease 2019 (COVID-19), caused by the novel severe acute respiratory syndrome coronavirus (SARS-CoV-2), has infected about 3 million people worldwide and killed more than 200,000, most being the elderly or people with potential chronic diseases or in immunosuppressive states. According to big data analysis, there are many proteins homologous to or interacting with the angiotensin-converting enzyme 2 (ACE2), which, therefore, may not be the only receptor for the novel coronavirus; other receptors may also exist in host cells of different species. These potential receptors may also play an important role in the infection process of the novel coronavirus. The current study aimed to discover such key proteins or receptors and analyze the susceptibility of different animals to the novel coronavirus, in order to reveal the transmission process of the virus in cross-species infection. We analyzed the proteins coded by the ACE2 gene in different mammalian species and predicted their correlation and homology with the human ACE2 receptor. The major finding of our predictive analysis suggested ACE2 gene-encoded proteins to be highly homologous across mammals. Based on their high homology, their possibility of binding the spike-protein of SARS-CoV-2 is quite high and species such as Felis catus, Bos taurus, Rattus norvegicus etc. may be potential susceptible hosts; special monitoring is particularly required for livestock that are in close contact with humans. Our results might provide ideas for the prevention and control of the novel coronavirus pneumonia.

Emerging infectious diseases (EIDs) pose a risk to global public health and biosafety. Over 5,000 viruses have been identified to date, of which ~75% are of a zoonotic origin, and can cross the species barrier and establish infection in human beings (1). Since December 2019, multiple cases of pneumonia of an unknown cause had been reported, which was subsequently identified as an acute respiratory infectious disease caused by a novel coronavirus infection, i.e., coronavirus disease 2019 (COVID-19) (2). Based on the results of genome comparisons, this novel coronavirus was named “severe acute respiratory syndrome coronavirus type 2” (SARS-CoV-2) by the International Committee on Taxonomy of Viruses, and was considered the primary pathogen of the current outbreak (3). The frequent and occasional regional outbreaks and uncertain epidemics have triggered serious social panic and caused huge economic losses, as the disease gradually spread globally. A previous study revealed the potential relationship between infection and history of contact with seafood and wildlife markets at the early stage (4). However, the source of SARS-CoV-2 has not been conclusively identified yet, since some patients did not have a history of exposure to wildlife markets at all.

Previous studies had documented infection from coronaviruses in humans, pigs, cattle, sheep, birds, dogs, cats, mice, camels, bats, and whales (5). Some hosts can be seriously infected with various coronaviruses, such as severe acute respiratory syndrome coronavirus (SARS-CoV) and the Middle East respiratory syndrome coronavirus (MERS-CoV). SARS-CoV-2 belongs to the β*-coronavirus* genus of the family *Coronaviridae*. The coronaviruses infecting human beings at present had originated from animals, and their natural hosts are generally Chiroptera (bats) and rodents (rats) (6). Additionally, different types of coronavirus can also infect Artiodactyla, including livestock (pigs, cattle, and camels), and carnivorous intermediate hosts, such as minks and civets (7). Whether SARS-CoV-2 can infect livestock (pigs and birds) and pets (such as dogs) is not yet clear. At present, there is insufficient understanding of the host-adaptive mechanisms of SARS-CoV-2, including the process of virus infection and replication, the function of virus coding proteins, interaction between the virus and its host factors, activation of the innate antiviral immune response of host, and the mechanism of viral escape from the host's immune system. Moreover, there is a lack of available approaches to deal with sudden viral infection events, to effectively target specific molecules to inhibit viral infection, and to treat the infection-related complications. In addition, with the source of the pathogen still being unclear, it significantly restricts extensive study and tracking of the route of transmission. Advancements in novel technologies could provide a new method to trace the source of the virus. Specifically, the possibility of suspect animals as intermediate hosts can be evaluated based on the binding characteristics of the viral proteins with different receptors. New technologies, such as artificial intelligence and shared data, are available for epidemiological investigation, thereby contributing to improved accuracy and screening efficiency.

Angiotensin-converting enzyme (ACE) is a monomeric, membrane-bound, zinc- and chlorine-dependent dipeptidase (8). It can catalyze the conversion of decapeptide angiotensin (Ang) I to octapeptide Ang II, and hydrolyze bradykinin by removing a C-terminal dipeptide (9). Angiotensin-converting enzyme 2 (ACE2), discovered as a homolog of ACE, functions as a carboxypeptidase that can preferentially cleave hydrophobic or basic amino acids at the carboxyl terminus. It can catalyze the conversion of Ang II to Ang-(1-7) and degrade Ang I to the inactive Ang-(1-9) (10). Ang-(1-7) is a vasodilator peptide with antioxidant, anti-fibrotic, and anti-inflammatory properties (11). ACE2 is highly expressed in the heart, kidneys, testis, hepatobiliary duct, and alveolar type 2 cells (12). Previous studies had predicted the structure of the spike-protein of SARS-CoV-2, and revealed it as a key protein that mediated virus invasion into host cells, interacted with ACE2 proteins, and mediated infection in humans (13).

The receptor binding domain of SARS-CoV-2 shares high sequence homology with SARS-CoV, indicating the potential binding of ACE2 with SARS-CoV-2 (14). The differences between SARS-CoV and SARS-CoV-2 were examined by electron microscopy. The results showed that SARS-CoV-2 binds to ACE2 with a higher affinity than SARS-CoV (15, 16). In accordance with the current data analysis, other species also have proteins with the same amino acid composition as the key region of the human ACE2 protein. This key region refers to the region that binds to the coronavirus spike protein. Other potential receptors may also exist in host cells of different species, which may play an essential role in the invasion of SARS-CoV-2. Therefore, besides humans and proven animals that can be infected, it seems imperative to analyze potential receptors in other species.

In this study, protein sequences corresponding to the ACE2 gene were downloaded from UniProt database (17), with subsequent construction of the phylogenetic tree, with the protein sequences, using the maximum likelihood method (18, 19). Figure 1 displays the distance distribution across ACE2 gene-encoded proteins in different species, with a high homology across those discovered in mammals.

**Figure 1:**
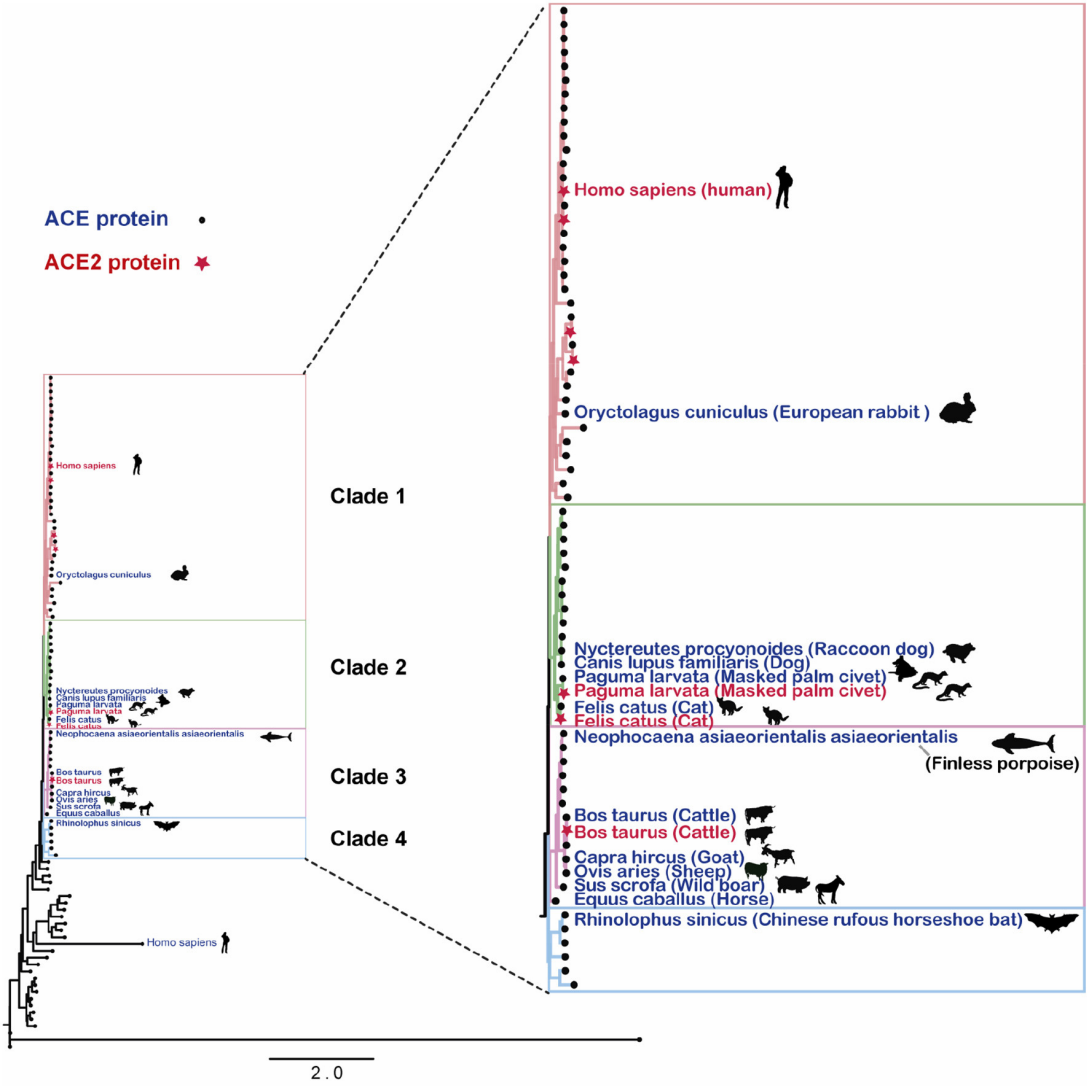
Angiotensin-converting enzyme 2 gene-encoded proteins in mammalian species. The RAxML tree was generated using RAxML-HPC2, with GAMMA model and a bootstrap value of 1,000 selected.

Shared data comparison was conducted, focusing on the key homologous proteins and core regions of different species. With the random selection of one species from each clade, further analysis was conducted on the crystal structure of N-terminal protease domain of ACE2 or key structural domains of other potential receptors and S-protein receptor-binding domain structure of SARS-CoV-2, so as to speculate the possibility of receptor-binding by SARS-CoV-2. Results indicated a high possibility of ACE2 binding to the S-protein of SARS-CoV-2 based on high homology (Figure 2). Superposition of the structural model of SARS-CoV-2 S-RBD complexed with ACE2/ACE from human, *Nyctereutes procyonoides* (Raccoon dog), *Neophocaena asiaeorientalis* (Finless porpoise), and *Rhinolophus sinicus* (Chinese rufous horseshoe bat) showed the complexes to have highly similar overall structures (Figure 2A). By analyzing the interacting residues between S-RBD and ACE2/ACE from different species in these complexes, two interacting regions (residues 19–84 and 346–360) were identified in ACE2/ACE. The sequences of these two regions from the species analyzed were found to be highly conserved (Figure 2B). However, the interaction interfaces between SARS-CoV-2 S-RBD and ACE2/ACE from different species in these complex structures were slightly different, with the ACE2 from humans having the maximum number of interacting residues, and being the largest buried area across the species (Figures 2C–F). This suggested ACE2 from humans could have a have higher affinity to SARS-CoV-2 S-RDB than those from other species.

**Figure 2:**
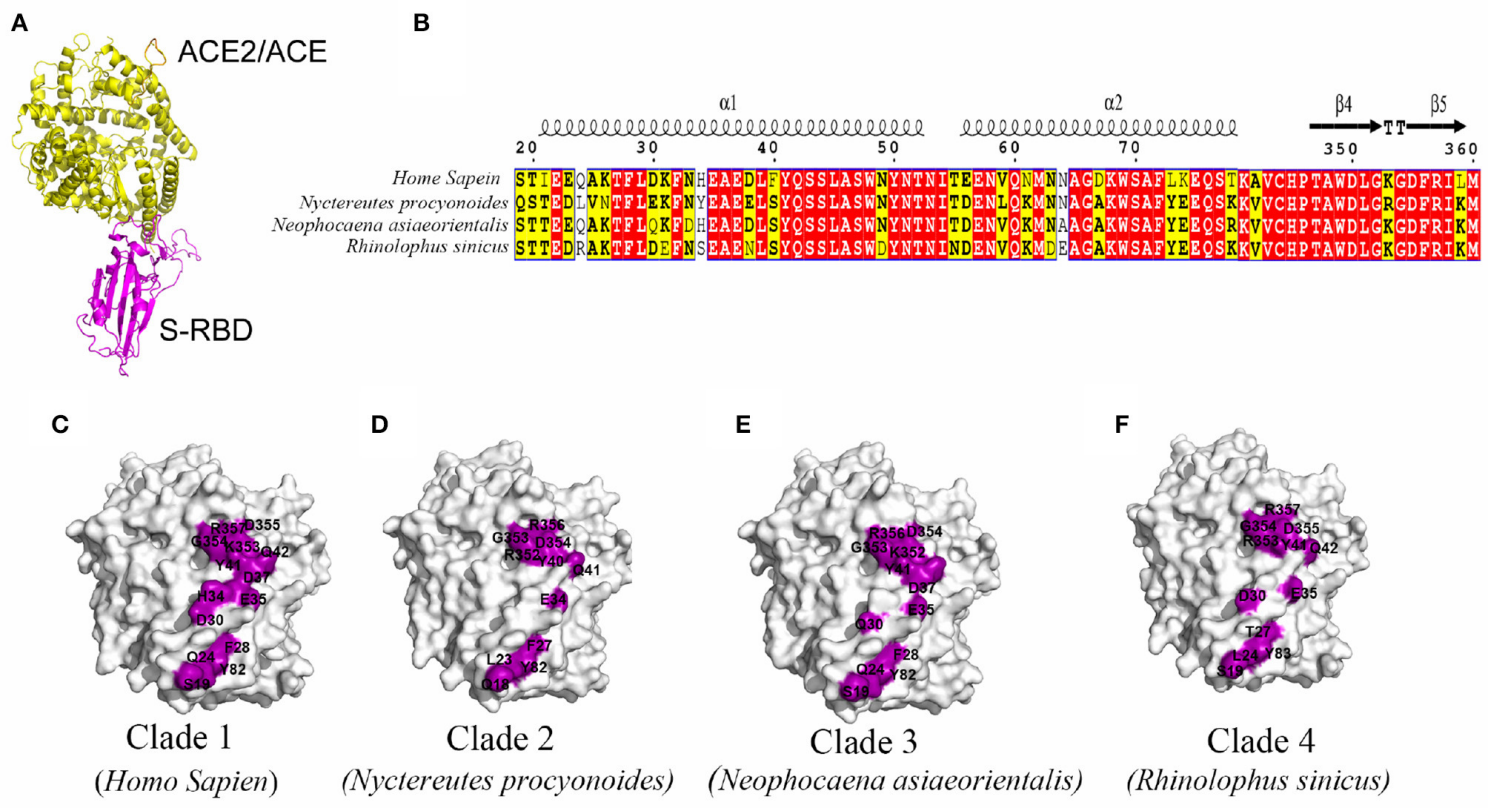
Prediction of S protein-binding domain structure of key domains in different species and severe acute respiratory syndrome coronavirus type 2 (SARS-CoV-2). **(A)** Superposition of the S-RBD in complex with ACE2/ACE (yellow) from human, *Nyctereutes procyonoides, Neophocaena asiaeorientalis asiaeorientalis*, and *Rhinolophus sinicus*. **(B)** Sequence alignment of two S-RBD binding regions (residues 19–84 and 346–360) in ACE2 from different species. **(C–F)** The interfacial residues (purple) in ACE2 (white) from human **(C)**, *Nyctereutes procyonoides* **(D)**, *Neophocaena asiaeorientalis* **(E)**, and *Rhinolophus sinicus* **(F)** that interact with S-RBD.

Due to different protein sequence lengths, in order to get better local sequence alignment, the Needleman-Wunsch algorithm was applied for the comparison with human ACE2 protein sequence and for the calculation of their similarities to study the amino acid composition distribution in key domains of each protein sequence (20). As shown in Figure 3, there was a high similarity of ACE2 gene-encoded proteins with the human ACE2 receptor, especially in the three domains bound to the S-protein of SARS-CoV-2. It consequently supported the higher potential susceptibility to infection in mammals.

**Figure 3:**
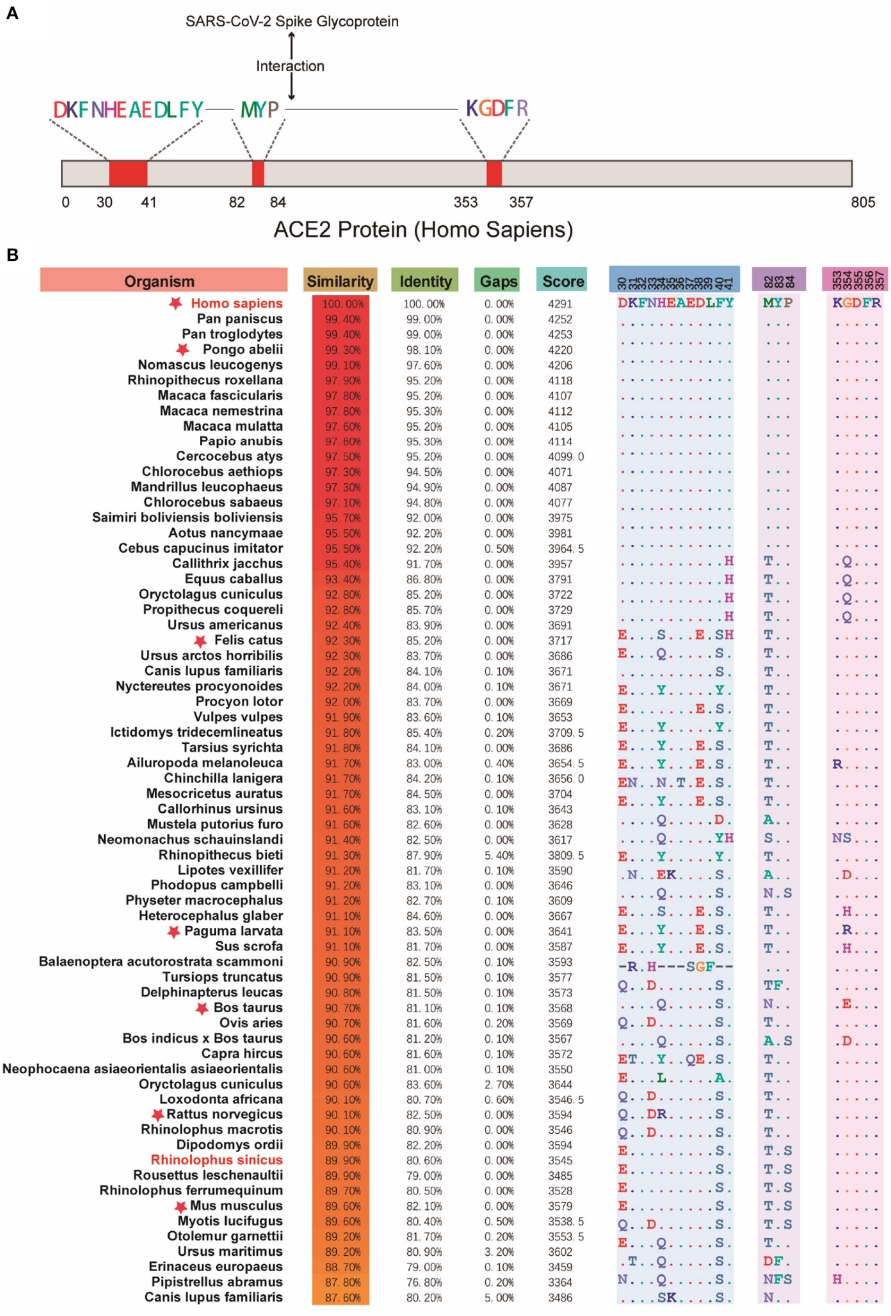
Comparison across the key domains of mammalian reservoir hosts. **(A)** The binding region of human ACE2 protein and SARS-CoV-2 virus S protein. **(B)** The results of the comparison between the key regions of proteins encoded by different mammalian ACE2 genes and human ACE2 protein. Red stars indicate the suspect species that deserves attention. The red stars indicate suspicious species that have been in close contact with humans or have been reported to be suspected of carrying SARS-CoV-2, such as Pongo abelii, Felis catus, Paguma larvata, Bos taurus, etc.

Furthermore, the binding ability of proteins encoded by different ACE2 genes and the potential receptor models for stimulating different species was analyzed. The interaction between ACE2 and SARS-CoV-2 was speculated to be the possible primary cause for the rapid spread of SARS-CoV-2. Compared with SARS-CoV, four of the five key residues of three short insertion and receptor binding sequences in the N-terminal region of SARS-CoV-2 were changed (21). Shi et al. had reported the replication of SARS-CoV-2 to be poor in dogs, pigs, chickens, and ducks, although it was quite efficient in ferrets and cats (22). They found SARS-CoV-2 to be transmitted across cats by respiratory droplets, the result consistent with homology comparisons (Figure 3). Other questions, regarding the binding ability of other potential receptors to viral proteins, a potential mutation that could further improve the interaction between S-protein and ACE2, or on species having highly homologous proteins or interacting with ACE2, remain to be addressed. Answers to these questions would facilitate the design of agents and antibodies against S-protein or ACE2 protein (or other potential receptors), or of small molecules, to disrupt their interactions.

In conclusion, the study of ACE2 gene-encoded protein products in mammalian species would be helpful to obtain more genetic and functional information about SARS-CoV-2. Based on their high homology, their possibility of binding the spike-protein of SARS-CoV-2 is quite high and species such as Felis catus, Bos taurus, Rattus norvegicus, etc. may be potential susceptible hosts; special monitoring is particularly required for livestock and poultry that are in close contact with humans. The potential susceptibility analyses of mammalian reservoir hosts, as well as the understanding of immune recognition and escape of the virus, would be of great significance for controlling the virus' spread, treating viral diseases, and protecting the life and property of people.

## Methods

### Data Collection and Phylogenetic Analyses

The protein sequences encoded by the ACE2 gene were downloaded from the UniProt database (15). If there were multiple identical protein sequences encoded by the ACE2 gene in each species, a sequence was randomly selected as the representative sequence of the species for subsequent processing. The screened sequences were aligned using Clustal Omega on the EBI web server (23). Maximum likelihood (ML) phylogenies of all viral genes were estimated by RAxML-HPC2 on XSEDE (18), with GAMMA model and a bootstrap value of 1,000 selected.

### Calculation of the Percent Identity of the Key Domains of Mammalian Reservoir Hosts

After screening, sequence similarity and identity were analyzed again to study further the relationship between the protein sequences encoded by the ACE2 gene. The key operation process can be divided into the following steps: The protein sequences from the source host that were not mammals were manually deleted, while the remaining protein sequences were compared with the ACE2 protein sequence encoded by the human ACE2 gene one by one using the Needleman-Wunsch algorithm (20), and the similarity and identity between them were obtained. Then, regions of the human ACE2 protein sequence that interacted with the severe acute respiratory syndrome coronavirus 2 (SARS-CoV-2) S proteins were highlighted and compared with the amino acid composition of protein sequences of other species.

### Prediction of S Protein-Binding Domain Structure of Key Domains

The SARS-CoV-2 S-RBD in complex with ACE2 from *Nyctereutes procyonoides, Neophocaena asiaeorientalis asiaeorientalis*, and *Rhinolophus sinicus* was modeled with Coot (24) using the crystal structure of the SARS-CoV-2 S-RBD in complex with human ACE2 (PDB ID: 6LZG) (15) as the template. The contact residues of the two partners in these modeled complex structures were determined with CoCoMaps server (25) with an atom contact distance cutoff of 4 Å.

## Data Availability Statement

Publicly available datasets were analyzed in this study. This data can be found here: https://www.uniprot.org/.

## Author Contributions

YC and JL conceived of the research, prepared the manuscript, and completed its revision. YC and YS performed data analysis. XT and ZB polished and finalized the figures. YG, WL, JQ, and DL provided various suggestions. All authors contributed to the article and approved the submitted version.

## Conflict of Interest

The authors declare that the research was conducted in the absence of any commercial or financial relationships that could be construed as a potential conflict of interest.
